# A Numerical Model of a Perforated Microcantilever Covered with Cardiomyocytes to Improve the Performance of the Microcantilever Sensor

**DOI:** 10.3390/ma14010095

**Published:** 2020-12-28

**Authors:** Bin Qiu, Guangyong Li, Jianke Du, Aibing Zhang, Yuan Jin

**Affiliations:** Smart Materials and Advanced Structure Laboratory, School of Mechanical Engineering and Mechanics, Ningbo University, Ningbo 315211, China; qiubin@nbu.edu.cn (B.Q.); zhangaibing@nbu.edu.cn (A.Z.); jinyuan@nbu.edu.cn (Y.J.)

**Keywords:** numerical model, microcantilever sensor, cardiomyocytes, contractile force

## Abstract

A few simple polymeric microsystems, such as microcantilever sensors, have recently been developed for the preliminary screening of cardiac toxicity. The microcantilever deflection produced by a change in the cardiomyocyte (CM) contraction force is important for understanding the mechanism of heart failure. In this study, a new numerical model is proposed to analyze the contractile behavior of CMs cultured on a perforated microcantilever surface for improving the performance of the microcantilever sensor. First, the surface traction model is used to investigate the bending displacement of the plain microcantilever. In order to improve the bending effect, a new numerical model is developed to analyze the bending behavior of the perforated microcantilever covered with CMs. Compared with the designed molds, the latter yields better results. Finally, a simulation analysis is proposed based on a finite element method to verify the presence of a preformed mold. Moreover, the effects of various factors on the bending displacement, including microcantilever size, Young’s modulus, and porosity factor, are investigated. Both the simulation and numerical results have good consistency, and the maximum error between the numerical and simulation results is not more than 3.4%, even though the porosity factor reaches 0.147. The results show that the developed mold opens new avenues for CM microcantilever sensors to detect cardiac toxicity.

## 1. Introduction

The heart is a vital human organ that circulates blood through the body by contraction and expansion. Quantitative analysis of the cardiomyocyte (CM) contraction amplitude and beating frequency is important for real-time monitoring of the toxicity of chemicals to myocardial cells, to help understand the mechanism of heart failure [1,2,3]. The CMs’ periodical beating has received more attention for the analysis of the biomechanical effects. Researchers have proposed some microsystems, such as microcantilever sensors [4,5,6], which can be used to investigate the contractile behavior of CMs cultured on the microcantilever surface. As a consequence, a change in contractility of the CMs can produce macroscopic bending behavior in the microcantilever [7,8,9]. Compared with the developed micropillar structure [10,11,12], the microcantilever approach does not need an optical microscopy image analysis and can easily be achieved by real-time analysis [13,14,15,16,17].

A microcantilever system usually requires optical microscopes to obtain the microscopy images or special strain gages integrated on the cantilever beam to detect the bending displacement produced by the CMs. The displacement of the microcantilever is very small when produced by the CMs. A slight increase in the displacement of the microcantilever can improve the detecting ability of the CMs’ contractile behavior. Although the microcantilever structure has some advantages compared to a micropillar, the bending effect should be improved to enhance the presence of the microcantilever sensors. Any geometrical shape change in the microcantilever structure can affect its mechanical efficiency. Nguyen et al. reported a perforated microcantilever that could decrease the microcantilever’s stiffness using a finite element method (FEM) simulation [18]. While some attempts have been made by researchers [11,12,13,14,15,16,17,18,19,20,21], the relationship between the contractile force generated by the CMs and the subsequent macroscopic behavior of a micro-mechanical device has not been investigated. Thus, further research needs to be performed on the influence of the structural parameters (such as porosity factor) on the bending effect of a perforated microcantilever.

In this work, a numerical model is presented to investigate the bending characteristics of a perforated microcantilever, where a layer of CMs has been cultured. It can improve the performance of the microcantilever sensors. In order to confirm the presence of mold, we proposed a finite element method (FEM) based on COMSOL Multiphysics software, which can be used to verify the presence of the proposed mold. Meanwhile, the effects of various factors on the bending performance, including the microcantilever size, Young’s modulus, and microcantilever’s porosity factor, are investigated. Finally, a transient analysis based on the time-varying contractile force is presented to validate the model.

## 2. Results and Discussion

### 2.1. Numerical Modeling of the Perforated Microcantilever

**Figure 1:**
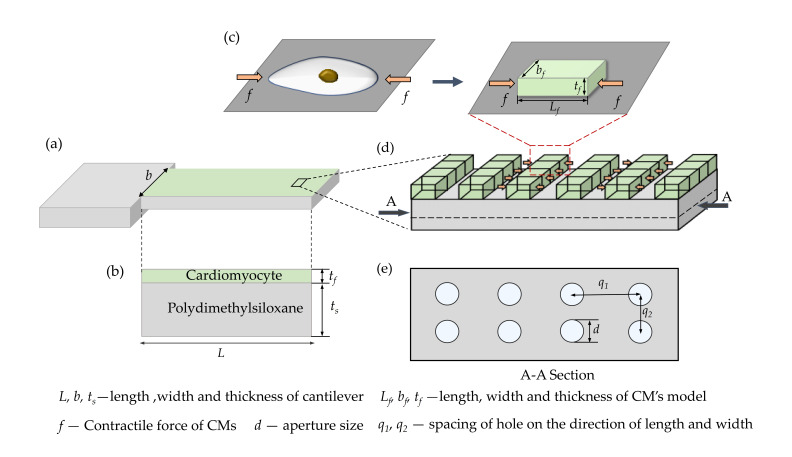
Molding of the perforated microcantilever covered with a layer of cardiomyocytes (CMs): (**a**) 3D structure of the perforated microcantilever covered with a layer of CMs; (**b**) side view of (**a**); (**c**) equivalent model of the CMs; (**d**) top view of (**a**); (**e**) a section of (**d**).

Figure 1 presents a 3D mold of the perforated microcantilever covered with a layer of CMs to improve the performance of the reported microcantilever sensors. The 3D structure of the perforated microcantilever, shown in Figure 1a, is composed of two layers. The bottom layer is the perforated microcantilever, and its size is *L* (length) * *b* (width) * *t_f_* (thickness). The top layer, with thickness *t_f_*, has CMs cultured on the surface of the microcantilever. The oval-shaped CMs are treated as cubes for a better understanding of the contractile force of the CMs, as shown in Figure 1c, whose length (*L_s_*), width (*b_s_*), and thickness (*t_s_*) are reported as 100 µm, 30 µm, and 10 µm, respectively [19,22]. Further, the contractile force in the other directions (except longitudinal direction) has little effect due to the vertical deflection of the microcantilever [23]. It is reported that the microgroove structures in the longitudinal direction can produce an accumulated action of the cultured CMs. However, when the CMs are arranged in the longitudinal direction, it can enhance the microcantilever’s macroscopic bending motion [24]. The CMs connect tightly to improve the microcantilever’s bending deflection, as shown in Figure 1d, with a magnitude of contraction of 2~5 nN/µm^2^ [25]. Figure 1e shows a perforated microcantilever section with a circular aperture, where *d* is the aperture size, and *q*_1_ and *q*_2_ are the spacing of the hole based on the direction of the length and width, respectively.

The CM layer consists of a thin film attached to the surface of the plain microcantilever. The contractile force of the CMs is equivalent to the residual stress of the thin film. Based on this model, Stoney’s equation is used to calculate the free-end deflection of the plain microcantilever, as shown in Equation (1) [26,27].

(1) $$\sigma_{f}=\frac{E_{s}t_{s}^{2}}{6\left(1-v_{s}\right)t_{f}}\left(\frac{1}{r}-\frac{1}{r_{0}}\right)$$

where r  and $r_{0}\;$ present the curvature radius of the substrate before and after bending; $E_{s}$ is Young’s modulus of the substrate; $v_{s}$ is Poisson’s ratio of the substrate; $t_{s}$ is the substrate thickness; $t_{f}$ is the film thickness; and $\sigma_{f}$ is the film stress, considered as the contractile force of the cardiomyocytes. Equation (1) presents the relationship between the substrate deflection and film stress. Berry conducted a more detailed mechanical analysis of the deformation of the coated cantilever and believed that it is more reasonable to replace the biaxial modulus *E_s_*/(1*−v_s_*) with the plate modulus *E_s_/*(1*−v_s_*^2^) of the substrates in the Stoney equation [28]. Thus, the modified Stoney equation can be expressed by the following equation:

(2) $$\sigma_{f}=\frac{E_{s}t_{s}^{2}}{6\left(1-v_{s}{}^{2}\right)t_{f}}\left(\frac{1}{r}-\frac{1}{r_{0}}\right)$$

Based on geometric theory, the relationship between the deflection of a plain cantilever and the curvature radius of the substrate can be achieved using Equation (3)

(3) $$r\approx \frac{L^{2}}{2\delta }$$

where *L* is the length of the cantilever; and *δ* is the vertical deflection of the cantilever. After substituting Equation (3) into Equation (2), the vertical displacement at the free end of a plain cantilever can be calculated by the following equation:

(4) $$\delta =\frac{3\left(1-v_{s}{}^{2}\right)L^{2}t_{f}\sigma_{f}}{E_{s}t_{s}^{2}}$$

Figure 1 presents a schematic of the perforated microcantilever to improve the bending effect. The reason is that the perforation can reduce the rigidity of the microcantilever. The substrate material of the plain microcantilever is considered a porous material that is dense. Several equations for the relationship between Young’s modulus and porosity have been reported [29,30], in which the most frequently used equation is as follows:

(5) $$E=E_{s}{\left(1-ap\right)}^{b}$$

where *E* presents the equivalent Young’s modulus of the porous materials; *E_s_* is Young’s modulus of the dense materials; *p* is the volume fraction of porosity (porosity factor); and a and b are constants and can be determined by experimental data fitting. By combining Equations (4) and (5), the following equation can calculate the vertical displacement at the free end of the perforated cantilever:

(6) $$\delta =\frac{3\left(1-v_{s}{}^{2}\right)L^{2}t_{f}\sigma_{f}}{E_{s}{\left(1-ap\right)}^{b}t_{s}^{2}}$$

In the present study, the experimental data can be obtained by using the FEM solution, whereas the relevant results are shown in Figure S1 (Supplementary Materials). The porosity factor, *p*, is decided by the aperture size and the hole spacing (*q*_1_ and *q*_2_). Constants a and b were calculated to be 0.49 and 5.86, using the MATLAB fitting tool.

### 2.2. Plain Microcantilever

**Figure 2:**
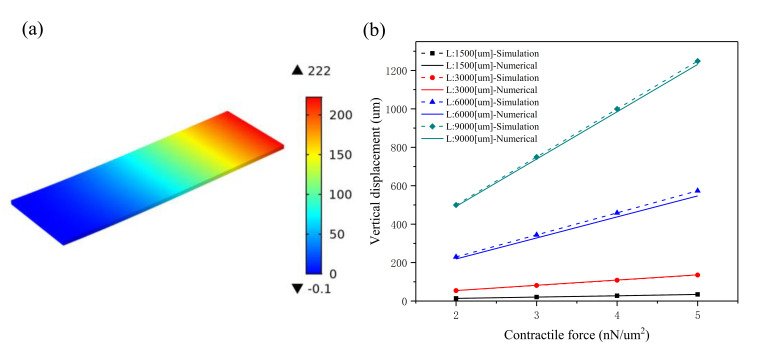
The numerical and simulation results of the maximum displacements at the free end of the plain microcantilever under different conditions (contractile force: 2~5 nN/μm^2^): (**a**) vertical displacement plot of the plain microcantilever (the microcantilever lengths are 1500, 3000, 6000, and 9000 μm, respectively) when the contractile force is 2 nN/μm^2^; (**b**) the effect of contractile force on the maximum displacements of the plain microcantilever with different sizes (the microcantilever lengths are 1500, 3000, 6000, and 9000 μm, respectively; the aspect ratio is 3:1; and thickness is 100 µm).

In order to confirm the efficiency of the proposed numerical model, the bending effect of the microcantilever without imperfection was first investigated by the modifier Stoney equation, as shown in Figure 2. Compared with the simulation results obtained by COMSOL Multiphysics software (COMSOL Multiphysics 5.4, COMSOL Inc, Stockholm, Sweden), the numerical results calculated by Equation (4) show that the modifier Stoney equation has a very high accuracy. The maximum deflection of the plain microcantilever under different conditions was studied, such as the size of the plain microcantilever, Young’s modulus of the substrate material, and the contractile force of the cardiomyocytes, studied based on numerical and simulation methods. Figure 2a shows the vertical displacement plot (obtained by COMSOL Multiphysics software) when the plain microcantilever lengths are 1500, 3000, 6000, and 9000 μm, respectively; the aspect ratio is 3:1, the thickness is 100 µm, and the contractile force is 2 nN/μm^2^. Here, Young’s modulus and Poisson’s ratio of PDMS (Polydimethylsiloxane) was found to be 750 kPa and 0.49, respectively [31]. Young’s modulus and Poisson’s ratio of the CMs are 188 kPa and 0.49, respectively [32,33]. Figure 2b compares the maximum displacements generated by the plain microcantilever with different sizes (the lengths are 1500, 3000, 6000, and 9000 μm; the aspect ratio is 3:1; and thickness is 100 um) when the contractile force is changed from 2 to 5 nN/μm^2^. The simulation results show that the maximum displacement of the plain microcantilever with a 1500 μm * 500 μm * 100 μm/3000 μm * 1000 μm * 100 μm/6000 μm * 2000 μm * 100 μm/9000 μm * 3000 μm * 100 μm size is 14 μm/54 μm/222 μm/500 μm, respectively, after a 2 nN/μm^2^ contraction force. When the contraction force is 5 nN/μm^2^, the maximum displacement of the plain cantilever reaches 34 μm, 135 μm, 574 μm, and 1249 μm, respectively. Compared with the simulation, the displacement generated by the numerical method is slightly lower; however, the two methods are consistent. A linear increase in the maximum bending displacement with an increase in contractile force is observed in our proposed numerical model. An error of both the numerical and simulation results may come from the thickness of the substrate, which is not much greater than the thickness of the film.

**Figure 3:**
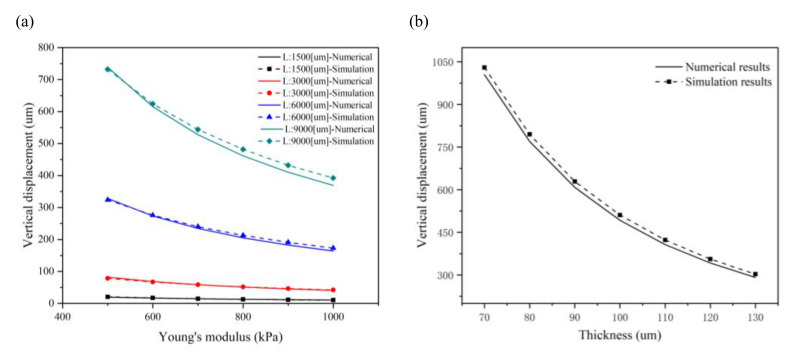
The numerical and simulation results of the maximum displacements at the free end of a plain microcantilever with different a Young’s modulus and substrate thickness: (**a**) the effect of Young’s modulus on the maximum displacement of the plain microcantilever with different sizes (the microcantilever lengths are 1500, 3000, 6000, and 9000 μm, respectively; the aspect ratio is 3:1; and thickness is 100 µm) when the contractile force is 2 nN/μm^2^; (**b**) the effect of substrate thickness on a maximum displacement when the microcantilever length is 9000 μm and the contractile force is 2 nN/μm^2^.

In order to further validate the accuracy of the present numerical method, the maximum displacements of the plain microcantilever with different substrates were characterized, as shown in Figure 3a. Here, Young’s modulus of the substrate material depends on the ratio of the PDMS base and curing agent. When the ratio is 10:1, Young’s modulus is 750 kPa, and the contractile force is 2 nN/μm^2^. Four sizes of a plain microcantilever are mentioned above. The simulation and calculated maximum displacement of a plain microcantilever is nonlinearly decreased when Young’s modulus is over 750 kPa. However, the rate of decrease in the maximum displacement slows down significantly. The simulation and numerical results are closer when Young’s modulus of the substrate material is lower, agreeing with the Stoney equation when Young’s modulus has a low value by assuming that Young’s modulus of the film and substrate are equal. Figure 3b shows the effect of substrate thickness on the maximum displacement when the microcantilever length is 9000 μm and the contractile force is 2 nN/μm^2^. The simulation and numerical results are in good agreement and show that the substrate thickness greatly influences the maximum displacement. When the thickness of the substrate is increased from 70 μm to 130 μm, with intervals of 10 μm, the maximum displacement is decreased from 1030 μm to 303 μm in the simulation analysis; however, for the numerical analysis, it is from 1005 μm to 291 μm.

### 2.3. Perforated Microcantilever

**Figure 4:**
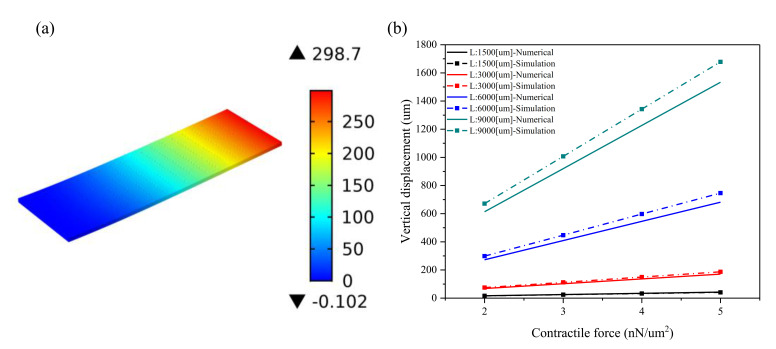
The numerical and simulation results of the maximum displacements at the free end of the perforated microcantilever under different conditions: (**a**) the vertical displacement plot of the perforated microcantilever when the contractile force is 2 nN/μm^2^ (*L* is 1500, 3000, 6000, and 9000 μm, respectively; *d* is 60 μm, *q*_1_ is 300 μm; and *q*_2_ is 100 μm); (**b**) the effect of the contractile force on the maximum displacements of the perforated microcantilever with different sizes (*L* is 1500, 3000, 6000, and 9000 μm, respectively; the aspect ratio is 3:1; *d* is 60 μm; *q*_1_ is 300 μm; *q*_2_ is 100 μm; and *t_f_* is 100 um).

The structure of a perforated microcantilever was covered with a layer of CMs to improve the bending effect. After designing a perforated microcantilever, the numerical model is proposed to investigate the bending effect. While the same size was proposed for a plain microcantilever, four different sizes were chosen for the perforated microcantilevers: *L* is 1500, 3000, 6000, and 9000 μm; aspect ratio is 3:1; *d* is 60 μm; *q*_1_ is 300 μm; *q*_2_ is 100 μm; *t_f_* is 100 μm; and d is 60 μm. The holes are regularly arranged on the microcantilever, and the number of holes in the four cases increases in proportion to ensure the same porosity. Figure 4 presents the numerical and simulation results of the maximum displacements at the free end of a perforated microcantilever under different conditions. Further, Figure 4a depicts a vertical displacement plot of the perforated microcantilever when the contractile force is 2 nN/μm^2^ (*L* is 1500, 3000, 6000, and 9000 μm, respectively; the aspect ratio is 3:1; d is 60 μm; *q*_1_ is 300 μm; *q*_2_ is 100 μm; and *t_f_* is 100 um). Since the aperture of the perforation is smaller than the size of the self-organized CMs, it is assumed that the perforation would not affect the arrangement and contractile force of the CMs. The perforated microcantilever has a lower stiffness due to holes in the body. The maximum displacement of the perforated microcantilever is observed at 299 μm, increasing by about 35% compared to a plain microcantilever with the same size, as shown in Figure 3a and Figure 4a. Figure 4b shows the effect of the contractile force on the maximum displacements of the perforated microcantilever with different sizes (*L* is 1500, 3000, 6000, and 9000 μm; the aspect ratio is 3:1; *d* is 60 μm; *q*_1_ is 300 μm; *q*_2_ is 100 μm; and *t_f_* is 100 um). When the contractile force of the CMs is increased from 2 to 5 nN/μm^2^, the simulation results show that the ranges of maximum bending displacement of the cantilever are 16~41 μm, 75~187 μm, 299~747 μm, and 671~1678 μm in the above four cases, respectively. Similarly, for the perforated microcantilever, the numerical results agreed well with simulation results using the FEM model in the four cases; however, the maximum bending displacement of the perforated microcantilevers is about 30% larger than the plain microcantilevers with lower stiffness. These results show the application potential of the perforated cantilever due to a higher bending displacement. Similarly, with the increase in contraction force, the maximum bending displacement increases linearly. For better visualization, 3D representation in Figure S2 regarding vertical displacement vs Contractile force.

**Figure 5:**
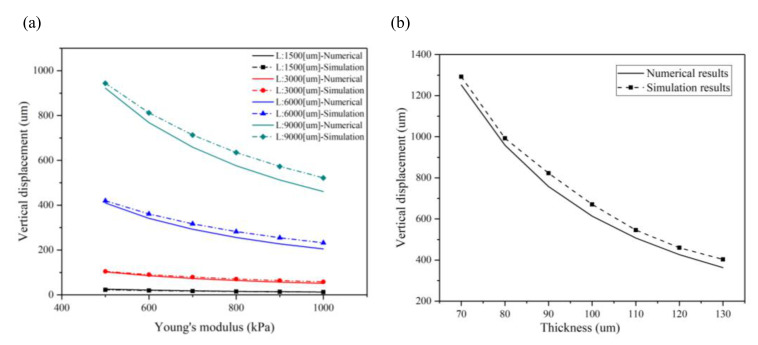
The numerical and simulation results of the maximum displacements at the free end of the perforated microcantilever with a different Young’s modulus and substrate thickness: (**a**) the effect of Young’s modulus on a maximum displacement of the perforated microcantilever with different sizes (*L* * *b* * *t_f_* is 6000 μm * 2000 μm * 100 μm; *d* is 60 μm; *q*_1_ is 300 μm; and *q*_2_ is 100 μm) when the contractile force is 2 nN/μm^2^; (**b**) the effect of substrate thickness on the maximum displacement when the microcantilever length is 9000 μm and the contractile force is 2 nN/μm^2^.

Young’s modulus on the bending displacement of the perforated cantilever was also examined, wherein Young’s modulus increased from 500 to 1000 kPa with intervals of 100 kPa, as shown in Figure 5a. Similarly, the numerical results agreed well with the simulation results, and both show a nonlinear, inverse relationship between Young’s modulus and bending displacement. However, when Young’s modulus exceeds 750 kPa, the bending displacement reduction rate decreases; the same tendency is also observed in a plain microcantilever. With an increase in Young’s modulus, the gap between the simulation and numerical results increases, as the larger the Young’s modulus of the substrate, the greater the error in Stoney’s calculation. Simultaneously, perforation can also further increase the bending displacement. Compared with the plain microcantilever, as shown in Figure 3a and Figure 5a, the maximum bending displacement in the simulation results shows an increase of 30%. In the numerical results, it offers an increase of 22% in all size cases. Therefore, the perforated microcantilever can obtain a lower bending displacement at a higher Young’s modulus. Furthermore, the effect of the perforated microcantilever thickness on the bending displacement was studied, as shown in Figure 5b, where the microcantilever length is 9000 μm and the CMs’ contractile force is 2 nN/um^2^. The numerical results agreed well with the simulation solution with an increase in the thickness of the substrate from 70 to 130 μm, with intervals of 10 μm. The maximum bending displacement decreased from 1292 to 403 μm in the simulation; however, in the numerical results, it is from 1224 to 355 μm. As we can see, the numerical results agreed well with the simulation results, and the thickness of the substrate has a significant effect on the bending displacement of the microcantilever. The perforated microcantilever has a larger bending displacement than the plain microcantilever; the maximum displacement increased by 21~33% compared with the plain microcantilever. Therefore, the perforated microcantilever can achieve a greater bending displacement on a thinner substrate. Due to the small Young’s modulus and the self-weight, the end of the microcantilever has a bending effect. Thus, the minimum thickness should be selected to ensure the stability of the structure in the experiment. In general, the error between the numerical and simulation results increases with an increase in microcantilever size, as shown in Figure 2 and Figure 4. For the plain microcantilever, the error between the numerical and simulation is a little higher than the other cases when the microcantilever length is 6000 µm, compared with the perforated microcantilever. The reason can be explained by the equivalent Young’s modulus and the porosity factor. As seen from Figure 4, the holes are regularly arranged on the microcantilever and the number of holes in the four cases increases in proportion to ensure the same porosity in theory. However, the porosity in the four cases is slightly different due to the constraint of the microcantilever size. When the microcantilever length is changed from 1500 µm to 9000 µm, the porosity is 0.094, 0.076, 0.068, and 0.065, respectively. For better visualization, 3D representation in Figures S3 and S4 regarding vertical displacement vs Young’s modulus and substrate thickness respectively.

**Figure 6:**
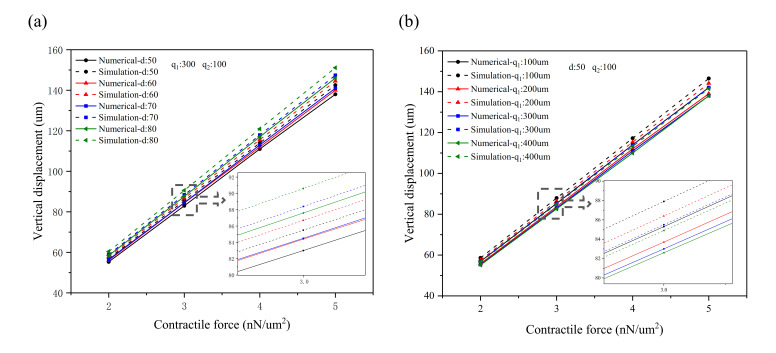
The effect of aperture and spacing on the bending displacement of a perforated microcantilever (*L* * *b* * *t_f_* is 3000 μm * 1000 μm * 100 μm): (**a**) the effect of aperture on the bending displacement (*q*_1_ is 300 μm; *q*_2_ is 100 μm); (**b**) the effect of spacing on the bending displacement (*d* is 50 μm; *q*_2_ is 100 μm).

In order to further study the influence of perforation on the bending displacement of the perforated microcantilever, the effect of aperture and hole spacing on displacement was characterized, as shown in Figure 6. Here, the length of the microcantilever is 3000 μm, the width is 1000 μm, and the thickness is 100 μm, whereas the contraction force is 2~5 nN/μm^2^. The proposed numerical model and FEM model were used to characterize the effect of different apertures and spacing on the bending displacement of a perforated microcantilever. The aperture, d, increased from 50 to 80 μm, with intervals of 10 μm, whereas the volume fractions of porosity were found to be 0.053, 0.076, 0.104, and 0.136, respectively (*q*_1_: 300 μm; *q*_2_: 100 μm), as shown in Figure 6a. When the contractile force of the CMs is 5 nN/μm^2^ and the aperture is 80 μm, the bending displacements were calculated to be 151.1 μm and 146 μm from the numerical and simulation solution, respectively. Figure 6b shows the effect of hole spacing on the bending displacement of a perforated microcantilever with an aperture *d* of 50 μm and *q*_2_ of 100 μm. The spacing *q*_1_ increased from 100 to 400 μm, with intervals of 100 μm, and the volume fractions of porosity were 0.147, 0.076, 0.053, and 0.041, respectively. When the contractile force of the CMs is 5 nN/μm^2^ and the spacing *q*_1_ is 100 μm, the bending displacements are 151.1 μm and 146 μm based on the numerical and simulation results, respectively. The ratio of increase in aperture and decrease in spacing can directly increase the porosity factor of a perforated microcantilever. It is assumed that porosity is an important factor affecting the stiffness of a perforated microcantilever. When the porosity factor increases to 0.147, Young’s modulus increases; however, the maximum error between the numerical and simulation results becomes 3.4% (*d*: 50 μm; *q*_1_: 100 μm; *q*_2_: 100 μm; contractile force: 5 nN/μm^2^), as presented in Figure 6. Therefore, the numerical results agreed well with the simulation results, confirming that the numerical model can work well.

### 2.4. Transient Analysis for Microcantilever Sensor

**Figure 7:**
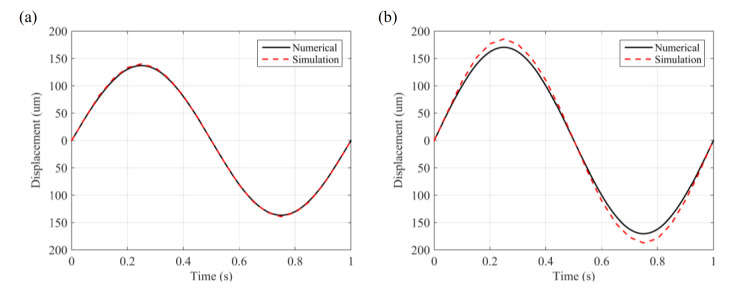
Displacement–time diagram of the transient analysis of the (**a**) plain microcantilever and (**b**) perforated microcantilever.

Figure 7 presents a transient analysis of a plain microcantilever and a perforated microcantilever to validate the feasibility of a numerical model for the microcantilever sensors. Here, the length of the microcantilever is considered to be 3000 μm, the width is 1000 μm, and the thickness is 100 μm. For the perforated microcantilever, *d* is 80 μm, *q*_1_ is 300 μm, and *q*_2_ is 100 μm. In order to simulate the contraction and expansion of the CMs, a time-varying contraction force based on a sine function was applied, as shown in the equation f=5sin(2πt), in which the period is 1 s and amplitude is 5 nN/μm^2^. Figure 7 presents the displacement–time function for a period of 1 s. The center point of the free end of the microcantilever is selected to obtain the displacement–time function diagram. With a period of 1 s, along with the change in contraction force, the displacement of the microcantilever reaches its maximum value at 0.25 s and 0.75 s, whereas the maximum displacement is almost identical in both the plain and perforated microcantilever, as observed in the static analysis. The displacement–time function of the microcantilever agreed well with the sine function in surface contraction, explaining that the displacement of the microcantilever directly reflected the transient change in surface contraction in the CMs. Besides, the perforated microcantilever has a higher displacement; the same results can be obtained from the transient analysis of the perforated microcantilever, as shown in Figure 7. Based on a static and transient analysis, our proposed model can detect the transient contractile force of CMs.

After the numerical analysis and simulation by COMSOL Multiphysics, the neonatal mouse ventricular cardiomyocytes (NMVCs) will be cultured on this type of perforated microcantilever to further validate the efficiency of the proposed numerical model in the next step. The microcantilever will be designed with a length (*L*), width (*b*), and thickness *(t_s_*) of 9000 μm, 3000 μm, and 100 μm, respectively, accompanied with holes whose diameter (*d*), length (*q*_1_), and width (*q*_2_) spacing are 60 μm, 300 μm, and 100 μm, respectively, uniformly distributed on the microcantilever. The PDMS prepared with the ratio of 10:1 PDMS base to curing agent will be chosen as the material to fabricate the microcantilever. To measure the displacement of the microcantilever, the end of the microcantilever is deposited onto an Au layer with a size of about 1000 μm in length, 1000 μm in width, and 100 μm in thickness. Therefore, the signal of the microcantilever bending can be detected with a laser amplifier. The accuracy of the proposed model in describing the bending effect can be further obtained by comparing it with the experimental values. We believe that the proposed numerical model of the perforated microcantilever is a precise model to forecast the bending effect and then to calculate the contraction force of the cardiomyocytes.

## 3. Conclusions

In this study, a new numerical model is used to investigate the bending of a perforated microcantilever sensor. We found that the numerical results were consistent with the simulation results. In the model, we considered the main force along the long axis of the microcantilever and ignored the other directions’ force, which has little effect on the vertical displacements of the cantilever. Based on the exert way of the CMs’ contractile force, the proposed numerical model was suitable for the microcantilever sensor with cultured self-organized CMs. As reported [23,24], when the CMs are cultured on the cantilever with microgrooves, the CMs exhibit a highly organized pattern and accumulation in the direction of the long axis of the microgroove. This feature will increase the vertical displacement of the cantilever, for easy observation with an optical microscope or detection by a strain gage. The proposed model based on this work condition, by changing the model factor, further improved the vertical displacement of the cantilever.

The effect of certain model factors, such as the contraction force of the CMs, microcantilever size, Young’s modulus, and porosity factor of the microcantilever’s behavior, were considered based on numerical and FEM methods. These influencing factors play an essential role in the mechanical performance of the microcantilever sensor. Studying the effect of these factors on a microcantilever has an important reference value for designing microcantilever sensors based on the same principle. Thus, the proposed model can more effectively analyze the bending displacement of the perforated microcantilever sensor. It is found that the mechanical response of a microcantilever directly reflect the transient change in the contraction of CMs, allowing us to understand the physiological function of self-organized CMs cultured on a microcantilever. Hence, it can efficiently detect the performance of CMs by monitoring the displacement of the microcantilever in real-time and has the potential for a high-throughput drug toxicity screening system in the future.

## Data Availability

Data available in a publicly accessible repository.

## Supplementary Materials

The following are available online at https://www.mdpi.com/1996-1944/14/1/95/s1, Figure S1: Fitted curve between equivalent Young’s modulus and the volume fraction of porosity, Figure S2: The numerical and simulation results comparison of the maximum displacements at the free end of the plain/perforated microcantilever under different conditions (contractile force: 2~5 nN/μm2), Figure S3: The numerical and simulation results comparison of maximum displacements at the free end of the plain/perforated microcantilever with different Young’s modulus, Figure S4: The numerical and simulation results comparison of maximum displacements at the free end of the plain/perforated microcantilever with different substrate thickness.
