# Supplementary material for: A Numerical Model of a Perforated Microcantilever Covered with Cardiomyocytes to Improve the Performance of the Microcantilever Sensor
Source: Materials (Basel). 2020 Dec 28;14(1):95. doi: 10.3390/ma14010095 (PMC7795391; doi:10.3390/ma14010095)
Supplement: Supplementary file 1 [file materials-14-00095-s001.pdf]

materials

Supplementary Material

A Numerical Model of a Perforated Microcantilever Covered with Cardiomyocytes to Improve the Performance of the Microcantilever Sensor

Bin Qiu, Guangyong Li *, Jianke Du *, Aibing Zhang and Yuan Jin

Smart Materials and Advanced Structure Laboratory, School of Mechanical Engineering and Mechanics, Ningbo University, Ningbo 315211, China; qiubin@nbu.edu.cn (B.Q.); zhangaibing@nbu.edu.cn (A.Z.); jinyuan@nbu.edu.cn (Y.J.)
* Correspondence: liguangyong@nbu.edu.cn (G.L.); dujianke@nbu.edu.cn (J.D.)

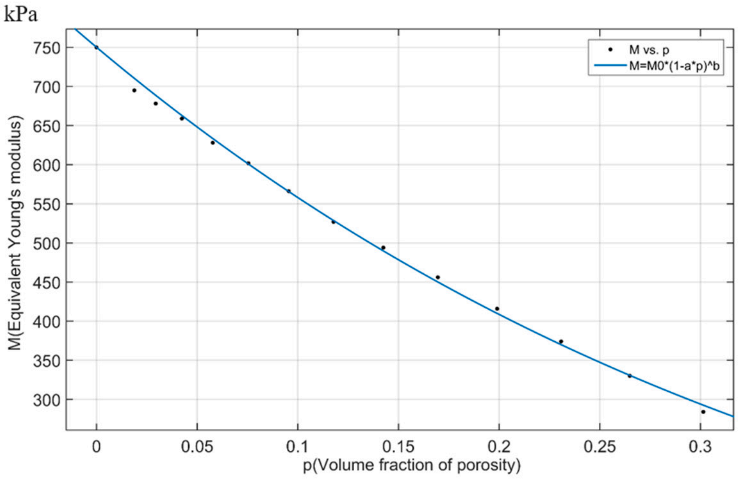

Figure 1. Fitted curve between equivalent Young's modulus and the volume fraction of porosity.

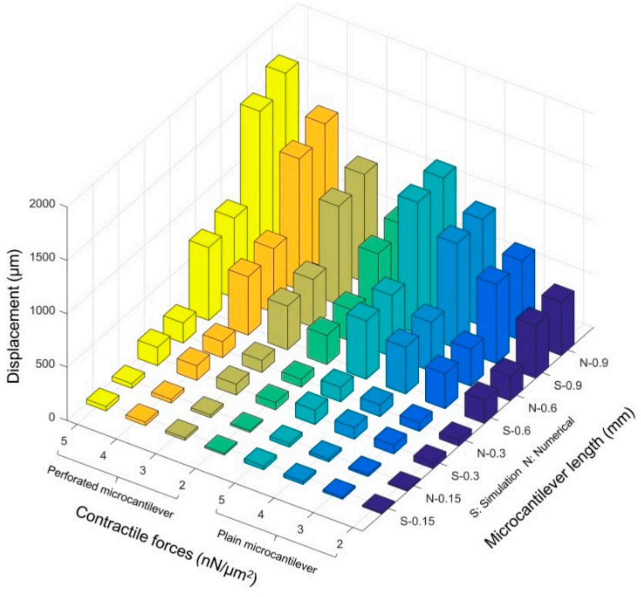

Figure S2. The numerical and simulation results comparison of the maximum displacements at the free end of the plain/perforated microcantilever under different conditions (contractile force: 2–5 nN/μm²).

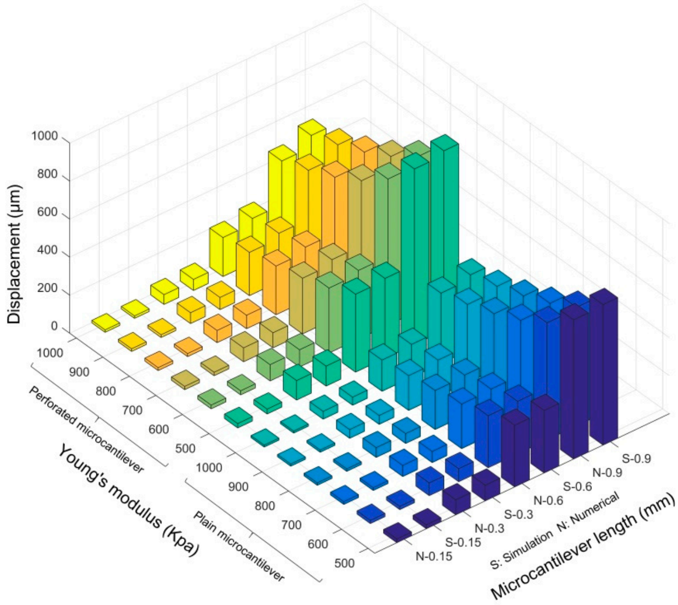

Figure S3. The numerical and simulation results comparison of maximum displacements at the free end of the plain/perforated microcantilever with different Young's modulus.

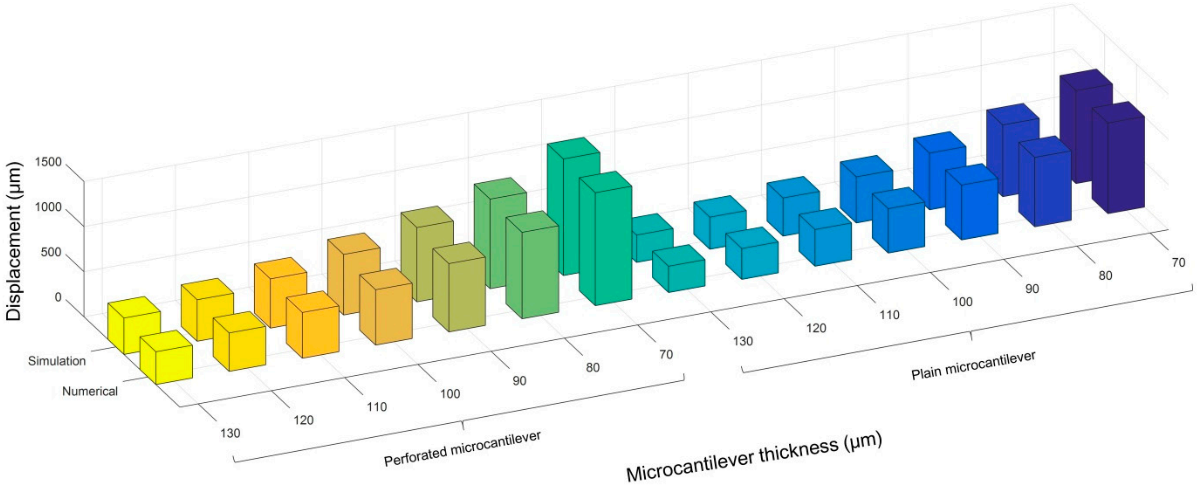

Figure S4. The numerical and simulation results comparison of maximum displacements at the free end of the plain/perforated microcantilever with different substrate thickness.
